# A Novel DNA Synthesis Platform Design with High-Throughput Paralleled Addressability and High-Density Static Droplet Confinement

**DOI:** 10.3390/bios14040177

**Published:** 2024-04-06

**Authors:** Shijia Yang, Dayin Wang, Zequan Zhao, Ning Wang, Meng Yu, Kaihuan Zhang, Yuan Luo, Jianlong Zhao

**Affiliations:** 1 State Key Laboratory of Transducer Technology, Shanghai Institute of Microsystem and Information Technology, Chinese Academy of Sciences, Shanghai 200050, China; yangshijia@mail.sim.ac.cn (S.Y.); dywang@mail.sim.ac.cn (D.W.);; 2 Center of Materials Science and Optoelectronics Engineering, University of Chinese Academy of Sciences, Beijing 100049, China; 3 School of Information Science and Technology, ShanghaiTech University, Shanghai 201210, China; 4 School of Microelectronics, Shanghai University, Shanghai 200444, China; 5 2020 X-Lab, Shanghai Institute of Microsystem and Information Technology, Chinese Academy of Sciences, Shanghai 200050, China; kzhang@mail.sim.ac.cn

**Keywords:** DNA data storage, DNA synthesis, static droplet, microfluidics, DRAM

## Abstract

Using DNA as the next-generation medium for data storage offers unparalleled advantages in terms of data density, storage duration, and power consumption as compared to existing data storage technologies. To meet the high-speed data writing requirements in DNA data storage, this paper proposes a novel design for an ultra-high-density and high-throughput DNA synthesis platform. The presented design mainly leverages two functional modules: a dynamic random-access memory (DRAM)-like integrated circuit (IC) responsible for electrode addressing and voltage supply, and the static droplet array (SDA)-based microfluidic structure to eliminate any reaction species diffusion concern in electrochemical DNA synthesis. Through theoretical analysis and simulation studies, we validate the effective addressing of 10 million electrodes and stable, adjustable voltage supply by the integrated circuit. We also demonstrate a reaction unit size down to 3.16 × 3.16 μm^2^, equivalent to 10 million/cm^2^, that can rapidly and stably generate static droplets at each site, effectively constraining proton diffusion. Finally, we conducted a synthesis cycle experiment by incorporating fluorescent beacons on a microfabricated electrode array to examine the feasibility of our design.

## 1. Introduction

The total volume of data generated by human society annually is experiencing exponential growth, gradually approaching the physical limits of existing storage media [1]. Under the current paradigm of solid-state storage, the demand for storing massive data imposes enormous pressure on material resources, personnel allocation, industrial power, and land usage [2,3]. DNA, with its exceptionally high data density and ultra-long storage lifespan, is considered a highly promising alternative as the next-generation storage medium [4,5,6,7,8]. The process of utilizing DNA for data storage mainly involves four steps: data encoding, DNA synthesis, DNA sequencing, and data decoding. Among them, DNA synthesis and DNA sequencing correspond to the writing and reading of the stored data, serving as the crucial foundational technologies in the entire storage process. DNA sequencing technology, benefiting from the enormous collective efforts from both academia (e.g., the Human Genome Project) and industry (including market giant Illumina and third-generation sequencing platforms like PacBio and Oxford Nanopore), has now surpassed DNA synthesis technology significantly in almost all relevant metrics including material cost, operation throughput, and time consumption [5,9]. Therefore, the current bottleneck hindering development in DNA data storage lies mainly in DNA synthesis and thus further advancement in high-density, high-throughput, and low-cost DNA synthesis technology is crucial for DNA data storage systems.

In the current DNA synthesis technological landscape, the most widely used platform is the conventional column synthesizers where a standard four-step reaction relying on the solid phase phosphoramidite chemistry is adopted [10]. Due to its low synthesis throughput (i.e., the number of unique oligonucleotides produced), high reagent consumption, and high costs for each synthesized oligonucleotide, the column synthesizers have failed to meet the increasing demands of evolving biological applications, let alone the more demanding requirement for data storage [5,11]. Chip-based high-throughput DNA synthesis strategies have been gradually developed over the past decades. Current high-throughput DNA synthesis methods still rely on the four-step cycle of phosphoramidite chemistry, with key modification in controlling the first deprotection step by incorporating inkjet printing [12], electrochemistry [13], photolithography [14], or other techniques [5,10] as means of paralleled DNA synthesis control at a massive scale. For instance, the inkjet DNA synthesizer involves the selective addition of nucleotides by directly spraying nucleotide monomer solution onto specified spots through an inkjet printing head [12]. While the inkjet DNA synthesizer is relatively straightforward, its site density is always constrained by the minimum droplet size that the printing head can generate and the positioning accuracy of the mechanical system. The light-directed DNA synthesis utilizes photolabile protecting groups in combination with photolithographic masks for selective deprotection [15]. The issue of higher cost induced by multiple masks was addressed by deploying the digital micromirror array for directing programmable light sources onto individual reaction spots [14]. During the synthesis, light scattering may lead to incorrect deprotection of neighboring sites around the illuminated spot, resulting in insertion errors. Therefore, light-directed synthesis faces challenges related to low synthesis quality and high synthesis costs. More recently, the light-directed platform has been applied in direct DNA data writing using >10,000 independent synthesis spots, with a specially designed error correcting algorithm to cope with the higher error rates, which is not suitable in biological applications requiring precision at the base-by-base level [16].

Most notably, electrochemically directed DNA synthesis involves selectively applying voltage to the reaction spots through an exposed electrode, initiating the electrochemical redox of hydroquinone/benzoquinone in an organic solvent. After the application of current to the microelectrodes, oxidation of hydroquinone near the anode occurs to generate protons, which removes the dimethoxytrityl (DMT) protecting group from the end of oligonucleotides. The excess protons will diffuse and continue to remove the DMT in the vicinity, until they approach the cathode and undergo a reduction reaction with benzoquinone to revert to hydroquinone [17]. The currently available electrochemically directed platforms report 90,000 independent synthesis spots with > 100-mer oligonucleotide arrays [18]. To restrict proton diffusion and interference among neighboring synthesis spots, Karl Maurer et al., from CustomArray Inc., introduced 2,6-lutidine as an organic base to the system for neutralizing excessive protons [13]. Recently, Nguyen et al. reduced the electrode diameter for electrochemically directed DNA synthesis to 650 nm, achieving a synthesis density of 25 million/cm^2^, with a specific aim at DNA data writing [11]. To confine proton diffusion in such a high-density electrode array, microwells of 200 nm depth were established around each electrode as physical shields, while simultaneously four cathodes were placed nearby each anode to reduce the diffusing protons through redox reactions.

**Figure 1:**
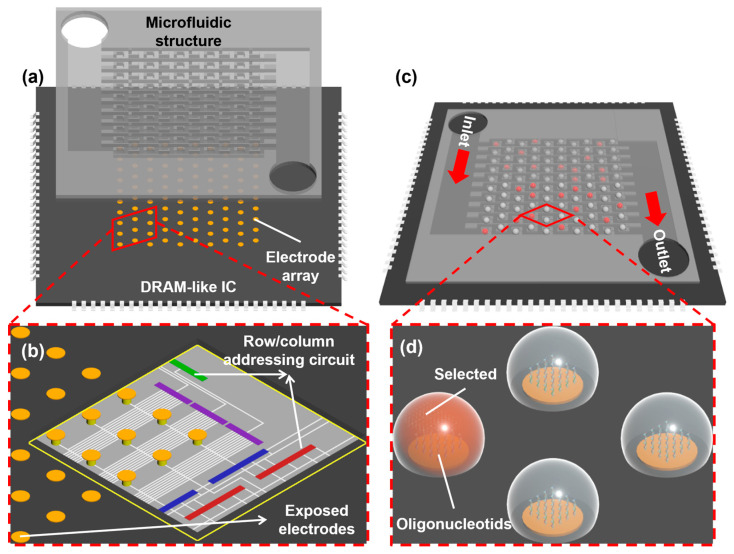
Schematic illustration of the overall design of a DNA synthesis chip and the synthesis process: (**a**) the DRAM-like IC and the SDA-based microfluidic structure. The enlarged view in the red dotted line box is (**b**) The electrode–IC connection. (**c**) The reagent forms independent droplets in a microfluidic structure and the IC selectively energizes sites to induce electrochemical reactions. The enlarged view in the red dotted line box is (**d**) The DMT at the oligonucleotide terminals is removed upon activation at selected sites and the protons are restricted in the droplet.

Among existing platforms, electrochemically directed DNA synthesis has achieved the highest density of synthesis spots. With the advancement of modern microelectronics design and fabrication processes, this method holds tremendous potential for even greater improvement in density and throughput. It could become the best candidate for high-speed data writing in future DNA data storage. However, as the number of individually addressable reaction sites expands (and thus the synthesis throughput), a more sophisticated circuit design is needed to implement a precise logic for controlling each electrochemical reaction. Moreover, as the density of sites increases, crosstalk caused by proton diffusion among neighboring sites continues to hinder the further development of this technology. In this study, we seek to explore the design of a novel high-density, high-throughput, electrochemically directed DNA synthesis platform (Figure 1). The presented platform utilizes a dynamic random-access memory (DRAM)-like integrated circuit (IC) to achieve individual addressing control of electrodes in high-throughput DNA synthesis. Simultaneously, it incorporates a static droplet array (SDA)-based microfluidic structure [19,20] to restrict proton diffusion, thereby avoiding strategies involving organic bases and cathodic reduction. Our design registers a throughput of 10 million possible synthesis spots with the density in the platform reaching 10 million/cm^2^, a combination that exceeds all reported high-throughput platforms to date [5]. In the following, we show the integrated circuit design for implementing an independent selection of electrodes and confirm its functionality in accurate addressability and freedom for voltage adjustment through simulation. We also demonstrate a static droplet formation using a two-phase flow microfluidic model. Finally, we prepared a microfabricated electrode array on which a preliminary oligonucleotide synthesis and characterization experiment was performed to test the feasibility of our design.

## 2. Methods

### 2.1. Integrated Circuit Simulation

The procedure for designing the integrated circuit unfolds as follows: initially, we crafted the Verilog code in line with the addressing specifications, subjecting it to pre-simulation for functional validation. Subsequently, we undertook a compilation phase to produce a netlist and scrutinized the logical consistency between the netlist and the code. Following this, the layout was crafted based on the generated netlist. Ultimately, post-simulation was executed post-extraction of the layout, subsequent to the clearance of DRC and LVS checks.

### 2.2. Microfluidic Numerical Simulation

The generation of droplets within the SDA-based microfluidic structure was simulated using the CFD module of COMSOL Multiphysics software (version 6.1), the interaction between dispersed and continuous phases in the two-phase flow was modeled using the level-set physics field. To reduce computational complexity and time consumption, simulations were conducted using a two-dimensional model. The continuous phase selected was FC-3283, with a dynamic viscosity of 1.365 × 10^−3^ Pa s and a density of 1.82 × 10^3^ kg/m^3^; while the dispersed phase was water from the COMSOL material library, with a contact angle set to 135° to simulate hydrophobic materials. To ensure accurate numerical simulation results in structures with significant size variations, a mapped mesh was employed to uniformly partition the simulation structure. The degree to which the mesh elements resemble the ideal geometric shapes—square in our 2D mapped mesh—is reflected in the average mesh element quality of 0.9948. A value closer to 1 indicates that the elements are well-shaped and size-consistent, which is essential for minimizing numerical errors and ensuring the reliability of the simulation outcomes. To mimic sequential fluid injection in experiments, an injection channel was placed before the SDA structure, with the SDA structure and the front inlet of injection channel pre-filled with the continuous phase, while the remaining portion of the injection channel was filled with the dispersed phase. The continuous phase was injected at a speed of 3 mm/s to ensure that when the array contained ten million capillary valve units with a density of 10^7^/cm^2^, the solution throughout the entire structure could be replenished within 10 s.

### 2.3. Microelectrode Array Fabrication

Electrodes were prepared on 4-inch silicon wafers with a silicon dioxide layer using a lift-off process, as shown in Supplementary Information (Supplementary Materials Figure S1). First, the wafers were baked at 150 °C for 5 min to remove moisture. Then, hexamethyldisilazane (HMDS) was spin-coated on the wafer at 2000 rpm for 10 s, followed by spin-coating of AZ NLOF 2020 negative photoresist at 500 rpm for 30 s and again at 2000 rpm for 10 s. After leveling, soft baking was performed at 100 °C for 90 s. The wafers were exposed for 10 s in the MA6B Double Sided Mask Aligner System, then post-baked at 100 °C for 90 s. Subsequently, the exposed wafers were immersed in ZX-238 developer for 2 min, then thoroughly rinsed with ultrapure water. A 20 nm titanium (Ti) adhesion layer and 70 nm gold (Au) layer were then deposited onto the wafer surfaces using a BC1800 Electron Beam Precise Nanoscale Deposition System. Finally, the patterned Ti/Au microelectrode arrays were obtained by lifting off the photoresist in acetone with ultrasonication.

### 2.4. Microelectrode Array Functionalization

The silicon wafer is initially sliced into chips, followed by a sonication process in acetone and IPA and thorough rinsing with ultrapure water. The microelectrode array chips undergo a 10 min cleaning process in a PDC-MG plasma cleaner at 150 W power. Hydroxyl groups are grafted onto the microelectrode array via electrografting of 4-aminophenylethanol. Initially, a 60 mL solution of 0.2M HCl is prepared at 0 °C and degassed in a vacuum pump for 5 min. Then, 41.1 mg of 4-aminophenylethanol is dissolved in the HCl solution, stirred thoroughly with a glass rod, and degassed for an additional 5 min. Next, 99.4 mg of sodium nitrite is added, ensuring complete dissolution. Then, degassing for an additional 5 min is carried out using a vacuum pump. The temperature of the solution is maintained at around 0 °C throughout the above procedures. The cleaned chips were immersed in a prepared solution, with the working electrode and working sense electrode of the Gamry 1010E electrochemical workstation clamped onto the pad of the chip. Functionalization of the electrode surface was performed using cyclic voltammetry, with a scan rate of 50 mV/s, starting and ending at the open-circuit potential (OCP), cycling 30 times between −0.5 and 0.8 V vs. Ag/AgCl. Finally, the electrodes were thoroughly rinsed with deionized water, acetone, and IPA and then dried for later use.

### 2.5. Oligonucleotide Synthesis and Characterization

First, 9.1 mg Cy3 was dissolved in 100 μL of activator to achieve a concentration of 0.1 M. The solution was injected into the microfluidic channel for 30 min by a syringe pump, ensuring thorough reaction of the activated Cy3 with the hydroxyl groups on the functionalized electrode surface. Subsequently, the channel was flushed with sufficient acetonitrile, followed by the injection of 1 mL of oxidizing agent for 2 min. Then, another round of acetonitrile flushing was carried out. The microelectrode array chip was immersed in acetonitrile, undergoing ultrasonic cleaning for 15 min to remove physical adsorption. The chip was subsequently removed, dried, and observed using the Zeiss LSM 900 laser scanning confocal microscope obtained from Oberkochen Germany.

## 3. Results

### 3.1. The Overall Design of the DNA Synthesis Platform

Figure 1 illustrates the overall design of the electrochemically directed oligonucleotide synthesis platform, which comprises a microelectronic chip with an exposed electrode array on the bottom layer and a microfluidic structure on the top. The exposed electrodes supply the required voltages for triggering proton generation and DMT deprotection, as well as serve as the solid supports for oligonucleotide molecules. An integrated circuit design is deployed underneath the electrode array to offer precise ON/OFF control of the voltage supplied to the electrodes. The microfluidic structure replaces the synthesis column in traditional DNA synthesizers, acting as a pathway for circulating DNA synthesis reagents. Simultaneously, the individual chambers in the microfluidic structure correspond one-to-one with the electrodes, covering them from above to restrict the diffusion of protons based on a static droplet formation mechanism (discussed later).

In this design, during the synthesis of oligonucleotides, the reagents sequentially flow through the chambers above the electrodes, and the oligonucleotide on the electrode surface undergoes multiple cycles of synthesis reaction to be extended base-by-base. The aqueous phase reagent flows through the microfluidic pathway and forms independent droplets in each chamber under the continuous non-aqueous phase flow. At this point, the DRAM-like IC selectively activates the electrodes for coupling a new batch of nucleotides, initiates voltage supply, and triggers the electrochemical reaction to generate protons to remove the DMT protective group from the terminal of the oligonucleotide on the electrode surface. Throughout the deprotection processes, the protons are confined within the droplets where the electrochemical reaction occurs and nearby oligonucleotides on idle electrodes are not affected. After completing each reaction, the IC ceases the voltage supply, and the droplets within each chamber are expelled through the channels. This process repeats until the desired oligonucleotide is synthesized.

### 3.2. The Dynamic Random-Access Memory-like Integrated Circuit Design and Simulation

**Figure 2:**
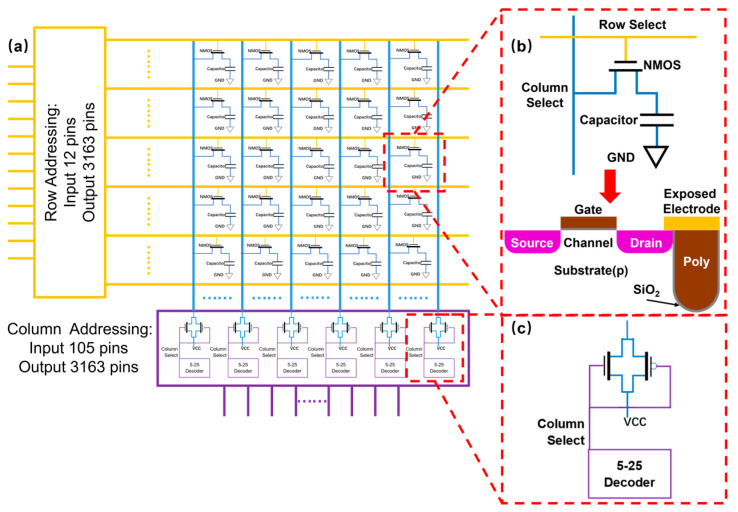
(**a**) The overall design architecture of the DRAM-like IC. (**b**) The basic unit of the DRAM-like IC consists of a transistor connected to a capacitor. (**c**) The column addressing circuit consists of 128 5-to-25 decoders.

The crucial functionality in our design is the ability to enable independently addressable voltage control in a massively paralleled manner. With the limitation of current electronic packaging technology, the number of input pins available for connection with peripheral apparatus is only around 2000–3000. Hence, in order to control the maximum number of electrodes with such limited available pins, we propose a DRAM-like integrated circuit design that is capable of achieving individual addressability for as many as 10 million electrodes with only 145 inputs. As shown in Figure 2a, the circuit includes two major modules: a row/column addressing circuits, and a two-dimensional function array with a total of 3163 × 3163 = 107 units. Each unit in the array is composed of a 1 pF capacitor connected to the drain of a field effect transistor (FET) (Figure 2b), with component layout dimensions specifically set at 3.16 × 3.16 μm^2^ (Supplementary Materials Figure S2), which corresponds to a density of 10 million units/cm^2^. The exposed electrode for nucleotide support also connects with the drain contact and directly acquires voltage input from the IC. In terms of controlling logic, firstly the row addressing circuit consists of a set of 12-to-3163 decoders. A 12-bit binary system has 212 independent encoding spaces, allowing it to represent integers ranging from 0 to 4095. For our array, only 3163 output ports are needed. Each output of the row addressing circuit is connected to the gates of all 3163 transistors in the same row.

When the code instruction selects a row to be powered on, the transistor gates of all the units within that row are at a high level, and thus the capacitance at the transistor drain is shorted to the output of the corresponding column addressing circuit. To improve efficiency and reduce the impact on circuit response time, the column addressing circuit uses a design that is different from the single-row selection of the row addressing circuit. It consists of 128 5-to-25 decoders, with a total of 133 input pins. The first 128 pins are used to control the working state of 128 decoders individually, while the last 5 ports serve as common inputs for all 5-to-25 decoders, as Mod (3163/128) + 1 = 25 (Figure 2c). Each of the 128 outputs is connected to the source of all 3163 transistors in the same column. This overall design of both row and column addressing circuits allows us to select a total of 128 units in a chosen row in one instance and another 128 cells from another row at the next instance.

As explained in the previous section, during each 4-step synthesis reaction cycle, one specific nucleotide base will be added to each selected spot. On average there are 10^7^/4 = 2.5 × 10^6^ units needed to be activated as only 1 out of 4 nucleotide bases will be incorporated. Hence, the working principle for our circuit design is as follows: the ~2.5 × 10^6^ units are divided into 19,532 groups of 128 each (Mod(2.5 × 10^6^/128) + 1 = 19,532). The IC will activate each group sequentially from first to last, cycle back to the first, and keep repeating this action. In one instance, 128 specific cells will be selected, meaning both their row and column outputs are at a high level (source and drain are connected, see Figure 2b).

The exposed electrode connected to the drain then receives a proper voltage signal and the corresponding capacitor will also be charged. Then, the IC switches to another 128 cells after the capacitor electrode reaches the required voltage values. In this way, even if these cells are not selected, their corresponding exposed electrodes maintain a certain voltage level and start a slow decaying process as the capacitors discharge themselves. From the electrochemical reaction perspective, the requirement is to maintain a relatively steady voltage level during the reaction cycle. Therefore, the key for all these components to function in a coherent manner is to ensure a fast charging and a slow discharging process for the capacitor. As long as the voltage drop on the capacitor is small enough after the IC logic cycles through all 19,532 groups and returns back, another charging process will resume and bring the voltage level back up. This entire IC control logic is run until the electrochemical reaction in all specified units is complete, and the next addressing cycle begins.

**Figure 3:**
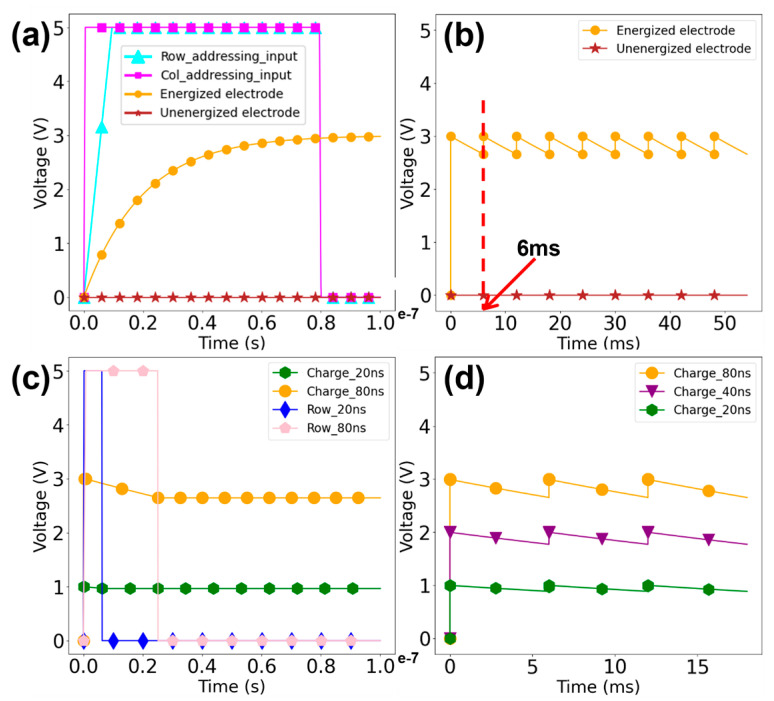
The post-simulation waveform: (**a**) The selected electrode (orange) is charged to 3 V in 80 ns and unselected electrodes (brown) are kept at 0 V with row/column addressing (cyan and magenta) input set at 5 V. (**b**) A relatively stable voltage output is maintained by the electrode through charge-discharge cycling, with a period of 6 ms indicated by the red dotted line. (**c**) Different charging times for different stabilization voltages. (**d**) The DRAM-like IC can precisely control the magnitude of the stable voltage within the 0–3 V range.

We first verify the addressing function of the integrated circuit, e.g., let us consider the example of addressing the synthesis site at row 666 and column 1111. Following the previously mentioned row/column addressing design and logic, we set the input of the row addressing circuit, which has 12 input ports, to 0b001010011010 (equivalent to 666). This action causes the decoder circuit to target row 666, thereby elevating all units on this row to a high voltage at their transistor gates. Subsequently, we set the 44th input among the first 128 inputs of the column addressing circuit, which contains 133 input ports, to a high level (5 V), effectively activating the 44th 5-25 decoder. We then set the 5 inputs of all the decoders to 0b01011 (equivalent to 11), leading the column addressing circuit to select the 44 × 25 + 11 = 1111th output port. With this configuration, the transistor at (666, 1111) is activated and charged. Observing the post-simulation waveform (Figure 3a), we notice that the unit at (666, 1111) is in a charged state (orange), while the voltage of other units is close to zero (brown), thus confirming the proper functioning of the addressing function.

Next, we seek to investigate whether our IC design can perform coherently and thus supply a relatively steady voltage supply for selected units. From the post-simulation waveform in Figure 3a, the capacitor reaches the designated voltage (3 V) within about 80 ns. Therefore, a full round of sequential charging for all selected units within one synthesis reaction, i.e., scanning through 3163 outputs in the row addressing circuit and cycling through 25 outputs of the 5-25 decoders in the column addressing circuit, would take a maximum time of 80 ns * 25 * 3163 = 6.3 milliseconds. Figure 3b shows the simulated waveform of repeating 80 ns charging and 6 ms discharging for one single unit. The voltage fluctuation is indeed within 5% of the peak level. Therefore, it can be considered that this cycle of charging and discharging ensures a relatively steady voltage supply for the electrode array during electrochemical synthesis reactions.

Furthermore, it is important to be able to adjust the voltage level according to the actual electrochemical environment. Specifically in our design, the supply voltage of the capacitor can be precisely adjusted by controlling the power-on duration of the column addressing circuit. As shown in Figure 3c,d, a longer charging period (from 20 ns to 80 ns) directly resulted in a higher voltage value (from ~1 V to ~3 V) at the capacitor. Such varying voltage values can be maintained for an extended amount of time using the logic described above. Our simulations indicate that with an external supply voltage of 5V, we could vary the output voltage level in a range of ~0–3 V (Figure 3d). This circuit design provides a sufficiently wide range of voltage adjustment, which can fully meet the changing voltage requirement of different electrochemical synthesis reactions [11,13,21].

### 3.3. The Static Droplet Microfluidic Design for Diffusion Prevention

**Figure 4:**
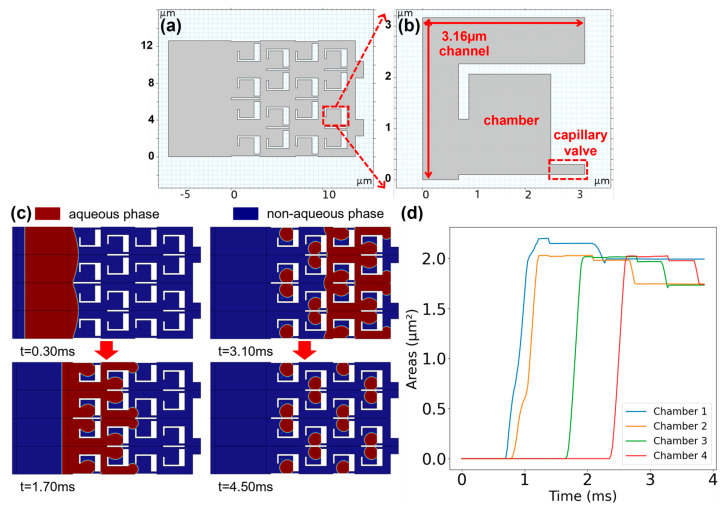
(**a**) The SDA-based microfluidic structure restricting the diffusion of protons in a high-density oligonucleotide synthesis array. (**b**) The enlarged view of capillary valve unit indicated by red dotted line including the bypass channel, capillary valve, and chamber (the reaction site). (**c**) The schematic of two-phase flow simulation results: the aqueous phase forms independent droplets in the reaction site driven by the oil phase. (**d**) The variation in the average areas of the aqueous phase within the chambers in the same column.

Another key issue for electrochemical deprotection is the interference of the generated protons affecting neighboring synthesis spots. We attempted to use an SDA-based microfluidic structure to restrict the diffusion of protons in a high-density array. As shown in Figure 4a, we employed a 4 × 4 array of SDA-based microfluidic structures. The array consists of replicated capillary valve units, with adjacent ones mirroring around the *x*-axis to achieve a more compact arrangement. We also adjusted the repeated units in the SDA-based microfluidic array to 3.16 × 3.16 µm to achieve an equivalent density of 10^7^ units/cm^2^, matching that of the IC. As shown in Figure 4b, each microfluidic unit comprises a bypass channel, a reaction chamber, and a capillary valve [22]. The bypass channel within the unit exhibits a 90-degree bend, connecting one end to the terminus of the preceding unit and the other end to the front of the subsequent unit. The capillary valve connects to both the current unit’s chamber and the bypass channel of the next unit, with the purpose of restricting the flow of the aqueous phase. The whole microfluidic structure is first filled with oil phase liquid and subsequently flushed with the aqueous phase. The aqueous phase liquid encounters significant flow resistance at the capillary valve entrance and thus is diverted through the bypass channel to the next unit. Additionally, the neck at the connection between the chamber and the bypass channel is constricted, making it narrower than the channel but wider than the chamber. This ensures effective filling of the chamber with the aqueous phase as it flows through. Simultaneously, when the aqueous liquid flows away from the bypass channel, the narrowed neck can sever the connected aqueous phase in the bypass channel and the chamber, allowing the liquid in the chamber to be retained and form a stable droplet.

Figure 4c shows a full sequence of aqueous phase static droplet formation using finite element simulation. Under forward liquid injection, the aqueous phase flows into the chamber and faces the resistance imposed by the capillary valve, causing the subsequent liquid to flow away through the bypass channel. When the aqueous phase in the channel separates from the chamber, the shear force at the neck connecting the chamber and the channel causes it to break into two parts. One part remains in the chamber to form an isolated droplet, while the remaining part flows into the next unit, repeating the process to form independent droplets in each chamber unit. Figure 4d illustrates the variation in the average areas of the aqueous phase within the rectangular chambers in the same column during the injection process. It can be observed that after the droplets in the chambers stabilize, except for slightly larger droplet areas in the chambers of the first column, reaching 1.99 square micrometers, the droplet areas in the chambers of the remaining columns stabilize around 1.74 square micrometers, exceeding half of the rectangular chamber area. The above simulation results indicate that during the rapid two-phase flow injection of the capillary valve unit array, the aqueous phase can automatically form independent droplets in the chambers above the sites. By using this microfluidic structure to confine proton diffusion, the protons generated by the electrodes can be completely restricted within the droplets, thereby avoiding crosstalk between sites.

### 3.4. Cyanine 3 Phosphoramidite Synthesis Experiment

**Figure 5:**
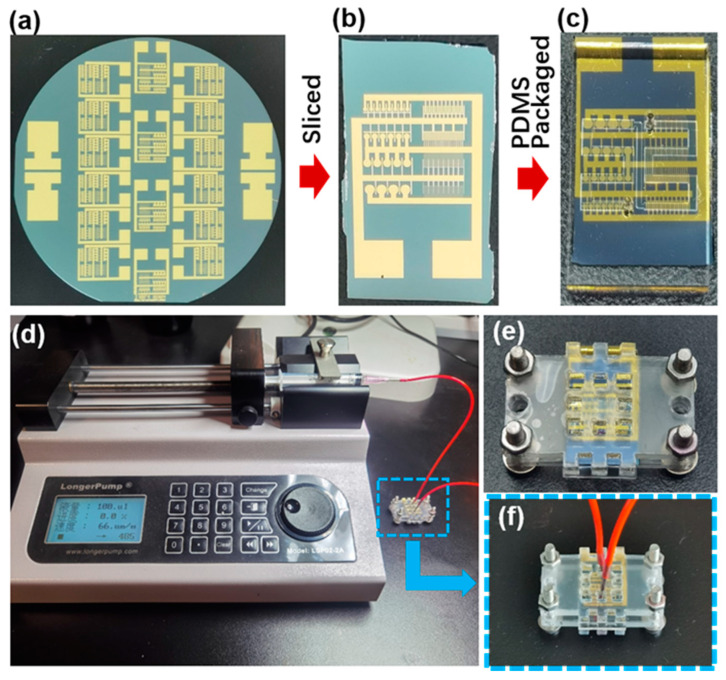
(**a**) The patterned Ti/Au microelectrode arrays on the silicon dioxide wafer. (**b**) The Ti/Au microelectrode arrays chip obtained by slicing the patterned silicon dioxide wafer. (**c**) The functionalized microelectrode array chip was aligned and attached to the PDMS microfluidic channel. (**d**) Cy3 synthetic reagents were injected into the microfluidic channel using a precision syringe pump (LongerPump LSP02-2A). (**e**) The chip and microfluidic channel were clamped together using customized PMMA fixtures and bolts. (**f**) Enlarged view of the chip in the blue dotted line box is during reagent injection.

**Figure 6:**
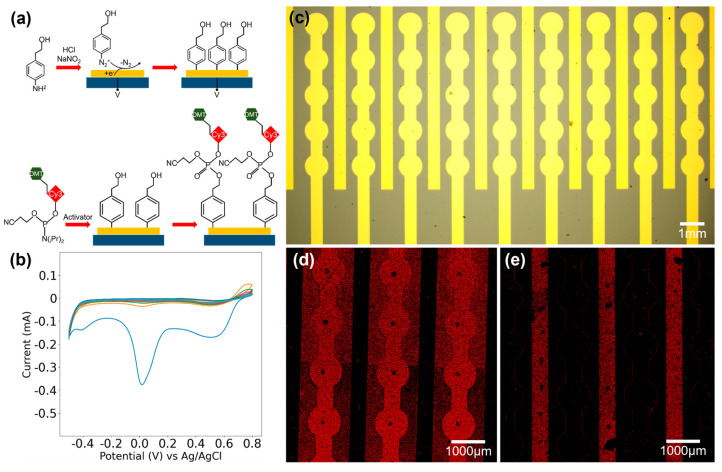
(**a**) Scheme of the electrografting and Cy3 phosphoramidite chemistry. (**b**) Cyclic voltammogram (CV) of 4-aminophenylethanol grafting on the Ti/Au electrode. (**c**) Microscopic photograph of the gold electrode array with Cy3 selectively synthesized on the (**d**) circular or (**e**) straight electrodes.

As a proof of concept, we set out to demonstrate the electrochemical control of adding fluorescent molecules on microfabricated gold electrode arrays. The chip packaging and experimental set-up are shown in Figure 5. Once the silicon wafer with gold electrode arrays was properly sliced into individual chips (Figure 5a,b), we further fabricated microchannels using polydimethylsiloxane (PDMS) that matched the microelectrode array chip for liquid injection to facilitate the cyclic addition of oligonucleotide synthesis reagents on the electrode surface and minimize costs (Figure 5c). The PDMS channels are adhered to the surface of the chip, sandwiched between two custom-made polymethyl methacrylate (PMMA) fixtures, and secured tightly using bolts to ensure a hermetic fit (Figure 5e). The reagent was administered into the device using a standard syringe pump (Figure 5d–f). To demonstrate how the selected electrodes can be activated through electrochemical methods, we first used the electrochemical reduction of diazonium salts, also known as electrografting, to achieve surface functionalization (Figure 6a). Figure 6b depicts the cyclic voltammogram (CV) of 4-aminophenylethanol grafting on the Ti/Au electrode, exhibiting a distinct reduction peak around 0.014 V. This reduction peak reaches the maximum value during the first scan (blue), gradually decreasing in subsequent scans (orange, green...), which is a typical feature of the diazonium reduction process. This phenomenon arises from the grafting of 4-(hydroxymethyl) benzenediazonium ions, derived from the reaction of 4-aminophenylethanol in a nitrous acid solution, onto the surface of the gold electrode. This grafting impedes electron transfer at the electrode surface and constrains subsequent diazonium reduction reactions.

Next, we chose to employ cyanine 3 (Cy3) phosphoramidite to represent a nucleotide during the synthesis cycle, and the reaction result was evaluated by the fluorescence signal on the electrode surface. This process used the same phosphoramidite chemistry as typical nucleotide incorporation. Figure 6c displays the electrode patterns on the microelectrode array chip, which contains two isolated types of electrodes: circular electrodes and strip electrodes. We functionalized only the circular electrodes or strip electrodes on separate chips, then injected Cy3 solution onto the chip surface. The solution then flowed over the circular and strip electrodes sequentially, resulting in coupling reactions. Figure 6d shows the synthesis results for the chip with functionalized circular electrodes only. Intensified red fluorescence can be clearly observed on the functionalized circular electrodes, while the untreated strip electrodes show no fluorescence. In contrast, Figure 6e displays fluorescence only on the functionalized strip electrodes of the chip, with no fluorescence on the non-functionalized circular electrodes. It is evident that only functionalized electrodes can couple with Cy3, while bare electrodes do not react with Cy3 and exhibit negligible physical adsorption.

## 4. Discussion

The electrochemically directed synthesis strategy offers great potential for achieving the highest density and throughput due to the advancement of microelectronics design and fabrication processes. Yet tremendous challenges still remain in pursuing high-throughput DNA synthesis, mainly in terms of individual voltage addressability with a growing number of synthesis spots and the proton interference among adjacent spots with increasing density. The number of independent input pins a chip packaging could offer is extremely limited compared to the amount that is needed for high-throughput DNA synthesis. We here repurposed the DRAM integrated circuit design previously used for memory chips to achieve the goal of independent addressability. Similar to memory chips, the electrochemical deprotection requires a steady voltage level to be maintained while being selected. As detailed in the previous section, the number of addressable electrodes scales exponentially with the available controlling input, leading to fairly manageable design constraints (i.e., 145 pins to control 10 million spots).

Another contribution in our design is the incorporation of the static droplet microfluidic structure as the restriction mechanism for preventing interference. Physical confinement is the most straightforward strategy in these circumstances. The major difficulty with this method is the fact that different reaction sites within the same chip cannot be completely isolated from each other. They need to share the same set of reagents that facilitate processes like deprotection, activation, oxidation, and flushing. Hence, as the size of each reaction site continues to shrink in order to pursue even higher density, it is becoming extremely difficult to rely only on partial physical confinement to prevent interference [23]. Two additional methods have been proposed, one deploying counter microelectrodes around each working electrode to act as a proton sink [11], and the other relying on dissolving organic base as a proton neutralizing agent [13]. Yet both methods could still become less effective as the density increases. The incorporation of static droplets aims to completely overcome such difficulties by using the oil phase to completely isolate each reaction site. In addition, even though the highest oligonucleotide synthesis density has been achieved by the method of providing both physical and chemical barriers by microwells and counter microelectrodes, more complicated IC design and processing are required to realize the individual addressing control of electrodes based on this mechanism in the future. It is also possible to use air as the non-aqueous phase and thus further simplify the reagent administration process during the synthesis experiment [24].

For the platform we designed, there are still some crucial issues that need to be addressed in platform development and implementation. Firstly, even though the state-of-the-art microelectronic manufacturing process can realize our design of a DRAM-like IC with 10 million electrodes on a 1 cm^2^ chip, the unconventional packaging is the most crucial component since it must be compatible with the electronic driving system and allow the IC chip and the microfluidic structure to bond together. Secondly, the PDMS surface has a serious adsorption issue, which can affect the efficiency of the synthesis. It is possible to further coat PDMS walls with SiO2 which has been recently reported to be able to eliminate non-specific adsorption and ensure maintenance of the synthesis [25]. In addition, because the minimum width of the microchannel (the width of the capillary valve) is 0.2 μm and bonding between the IC chip and the microfluidic chip needs to be considered, our microfluidic design cannot be easily realized with PDMS materials. On the other hand, the utilization of glass as the structural material for microfluidics can potentially address both issues and result in further improvement of the device performance.

## 5. Conclusions

In summary, this work presents a novel design of a DNA synthesis platform targeting high-throughput DNA data writing for DNA data storage equipped with a DRAM-like circuit and SDA microfluidics. The integrated circuit enables individual addressability of the electrodes in high-throughput electrochemically directed DNA synthesis, showing significant potential for increasing synthesis density and throughput. The corresponding SDA-based microfluidic structure features an array of repeating capillary valve units. By employing a two-phase flow driving mechanism, independent liquid droplets can form above the electrode unit during the liquid introduction process. Electrochemically generated protons at the sites during the reaction cycles are confined to individual droplets, thereby completely shutting off diffusion to other sites and any possible reagent interference. Based on the simulation verification, our IC design can achieve individual addressing and voltage control of as many as 10 million electrodes. It allows flexible adjustment of the voltage magnitude within a certain range based on the actual requirements of the electrochemical reaction. Simultaneously, the microfluidic design enables the rapid generation of static droplets at high flow rates and in a short time. Compared to existing platforms, our newly designed electrochemically directed DNA synthesis chip can achieve a synthesis density of 10^7^/cm^2^, with the potential for further improvement. Furthermore, we conducted oligonucleotide synthesis experiments. Fluorescence images showed that we successfully synthesized Cy3 on a gold microelectrode array, laying the foundation for future implementation.

## Data Availability

The data that support the findings of this study are available from the corresponding author upon reasonable request.

## Supplementary Materials

The following supporting information can be downloaded at: https://www.mdpi.com/article/10.3390/bios14040177/s1, Figure S1: Schematic diagram of the manufacturing process for microelectrode array; Figure S2: (a) The layout of DRAM-like IC design. (b) Three partial enlarged views of the layout showing detailed components in row/column addressing logic circuit and the two-dimensional array.
