# Supplementary material for: A Novel DNA Synthesis Platform Design with High-Throughput Paralleled Addressability and High-Density Static Droplet Confinement
Source: Biosensors (Basel). 2024 Apr 6;14(4):177. doi: 10.3390/bios14040177 (PMC11047993; doi:10.3390/bios14040177)
Supplement: Supplementary file 1 [file biosensors-14-00177-s001.zip › biosensors-2926172-supplementary.pdf]

Supporting Information

High-density DNA synthesis chip with integrated circuit addressing and static droplet confinement

Shijia Yang, [1,2†] Dayin Wang,[1,2,3†] Zequan Zhao,[1,2] Ning Wang,[1,2,3] Meng Yu,[4] Yuan Luo,[1,2*] and Jianlong Zhao[1,3*]

[1]State Key Laboratory of Transducer Technology, Shanghai Institute of Microsystem and Information Technology, Shanghai, 200050, P. R. of China

[2]Center of Materials Science and Optoelectronics Engineering, University of Chinese Academy of Sciences, Beijing, 101408, P. R. of China

[3]School of Information Science and Technology, ShanghaiTech University, Shanghai, 201210, P. R. of China

[4]School of Microelectronics, Shanghai University, Shanghai, 200444, P. R. of China

[†]These authors contributed equally: Shijia Yang and Dayin Wang.

[*]Author to whom correspondence should be addressed: yuanluo@mail.sim.ac.cn, jlzhao@mail.sim.ac.cn

This PDF file includes:

Supplementary Note 1
Supplementary Figures S1 & S2

Supplementary Note-1

We designed a row/column addressing circuit to achieve independent addressing of 10^7 units (Fig. S2). The electrode array units consist of nMOS-connected capacitors, with a size of 3.16×3.16 μm². The row addressing circuit has 12 input ports and 3163 output ports, connected to the nMOS gates. The column addressing circuit has 133 input ports and 3163 output ports, connected to the nMOS sources, enabling 128 parallel addressing controls.

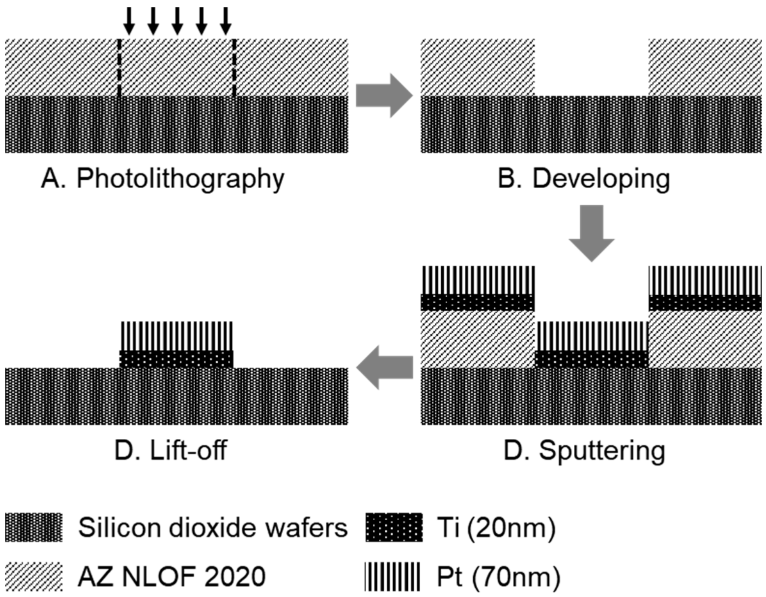

Figure S1. Schematic diagram of the manufacturing process for microelectrode array.

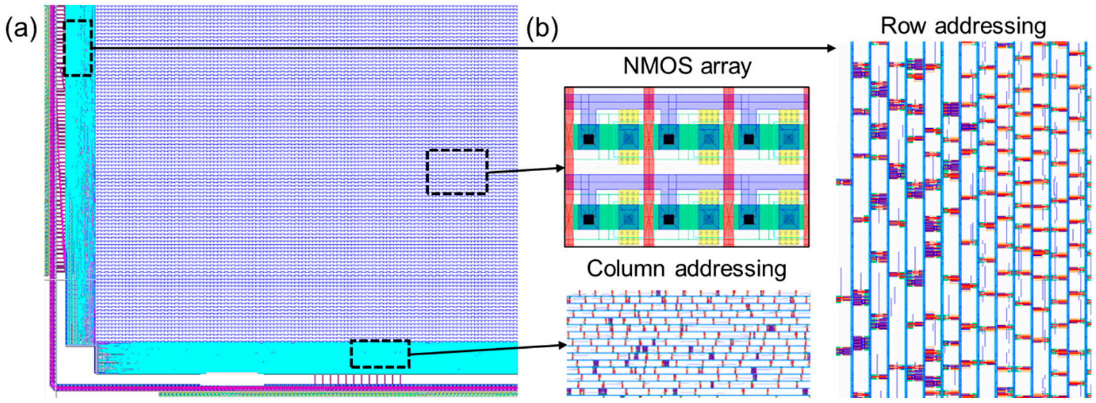

Figure S2. (a) The layout of DRAM-like IC design. (b) Three partial enlarged views of the layout showing detailed components in row/column addressing logic circuit and the two-dimensional array, each unit within the array is $3.16 \times 3.16 \mu m^2$.
